# Rapunzel syndrome: an infrequent cause of severe iron deficiency anemia and abdominal pain presenting to the pediatric emergency department

**DOI:** 10.1186/s12887-018-1097-8

**Published:** 2018-04-04

**Authors:** Giuseppe Cannalire, Luigi Conti, Maurizio Celoni, Carmine Grassi, Andrea Cella, Giulia Bensi, Patrizio Capelli, Giacomo Biasucci

**Affiliations:** 1 Department of Pediatrics and Neonatology, Guglielmo da Saliceto Hospital, Cantone del Cristo 50, Piacenza, Italy; 2 Department of General, Thoracic and Breast Surgery, Guglielmo da Saliceto Hospital, Cantone del Cristo 50, Piacenza, Italy

**Keywords:** Rapunzel syndrome, Abdominal pain, Iron deficiency anemia, PICA, Trichobezoar, Trichophagia, Trichotillomania, Case report

## Abstract

**Background:**

Iron deficiency anemia (IDA) and abdominal pain are commonly seen in a pediatric emergency department (8 and 18% incidence respectively in our center). They are manifestations of a wide variety of diseases ranging from benign to immediately life-threatening. Trichobezoar is an under-diagnosed entity that has to be considered in children and adolescents, expecially female, suffering from trichotillomania (compulsion to pull hair) and trichophagy (compulsion to swallow hair). When undiagnosed, gastric bezoars may cause gastric ulceration, perforation, haemorrhage and obstruction.

**Case presentation:**

To underline the importance of including this pathology in the differential diagnosis of IDA and abdominal pain, we present the case of a 14 year-old girl with a huge trichobezoar which completely filled the stomach and extended into the small bowel. Since trichobezoar has an extension to the small bowel, it is classified as Rapunzel syndrome. As the bezoar couldn’t be removed by endoscopy, the girl underwent surgical intervention. The patient passed through a gradual re-feeding, with iron and vitamins supplementation, and through a psychiatric counselling.

**Conclusion:**

The Rapunzel syndrome is a rare entity that may be complicated by life-threatening events. A prompt diagnosis and an appropriate therapy can reduce comorbidities. Gradual re-feeding with supplementation of micronutrients allows adequate catch-up weight with normalization of haematochemical nutritional parameters. Since many of these patients suffer from psychiatric pathology such as PICA with emotional problems and mental retardation, psychological/psychiatric counselling plays an important role in order to prevent bezoar recurrence.

## Background

Bezoars are collection of non-digestible foreign bodies such as hair that usually accumulate in the gastrointestinal tract. They are commonly found in domestic animals, whereas in humans it is a rare condition mainly seen in female adolescents [1]. In the absence of adequate treatment, associated mortality rate is up to 30%, primarily because of gastrointestinal bleeding, destruction or perforation [1, 2].

Trichobezoars are more commonly found in children and adolescents with normal gastrointestinal function and usually result from an underlying behavioral disorder known as “Rapunzel syndrome” [3] which is characterized by the presence of a gastric foreign body, usually hair, extending from stomach into the small bowel: it often occurs in young patients suffering from trichotillomania (compulsion to pull hair) and trichophagy (compulsion to swallow hair). The mass may not cause any symptoms until it becomes too large, because of the high capacity of the stomach.

Symptoms of the obstruction are recurrent abdominal pain, nausea, vomiting, anorexia, weight loss and malabsorption of trace elements. When undiagnosed, gastric bezoars may also cause severe anemia either due to malabsorption or gastrointestinal bleeding [4].

Methods of removal of small gastric bezoars are minimally invasive, such as endoscopy but large trichobezoars, which often are non-fragmentable, can only be removed by open surgical intervention.

Our report describes a trichobezoar, classified as Rapunzel syndrome, in a 14 year-old girl and highlights the salient features of diagnosis and treatment.

## Case presentation

A 14 years old girl was brought to our pediatric emergency unit because of severe hypocromic microcytic anemia detected by her primary care physician who had required blood tests to investigate the cause of reported asthenia and weight loss. Her mother reported that the patient had experienced early satiety and abdominal pain for several months. No history of fever or trauma as well as no dysuria, gross hematuria or recent travel abroad. Reported regular menstrual cycles until 2 months before admission.

With regards to the past medical history, a neurosurgical intervention for cranial meningocele of the bregmatic region at the age of 6 years and laparoscopic cholecystectomy for gallstones at the age of 13 years have been reported. At the time of cholecystectomy, hemoglobin was normal as well as hemoglobin electrophoresis. Initially, parents denied that their daughter had any history of eating disorder such as PICA, though alopecia was reported.

At physical examination: anorexic habitus, pulse rate of 88 beats/min, respiratory rate of 28/min, very pale skin and mucosae, no evident bleeding nor icterus. Her abdomen was rigid under palpation with a non- compressible mass in the left upper quadrant.

Laboratory tests, including erythrocyte morphology, confirmed a severe IDA, with normal Serum electrolytes, amylase, thyroid and liver function tests and vitamin B6, B12 and folate at the lower range of norm. Fecal occult blood test was positive.

**Fig. 1:**
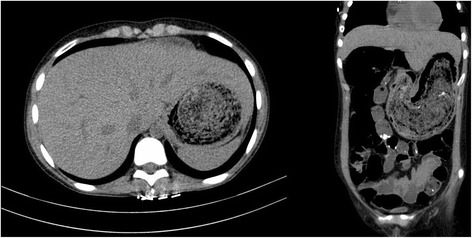
CT, Coronal and sagittal view: huge, well defined, multi-layered, heterogeneous, solid appearing, mass filling the gastric fundus and antrum and extending into duodenum. Since trichobezoar has an extension to the small bowel, it is classified as Rapunzel syndrome

Abdominal ultrasound (US) showed perihepatic free fluid and mild splenomegaly (10.5 cm diameter) and computed tomography (CT) scan reported severe gastric dilatation due to organic matter accumulation. The mass completely filled the gastric fundus and antrum and extended into duodenum. Also, small bowel dilatation and air-fluid levels were present (Fig. 1). A gastric bezoar was therefore suspected.

**Fig. 2:**
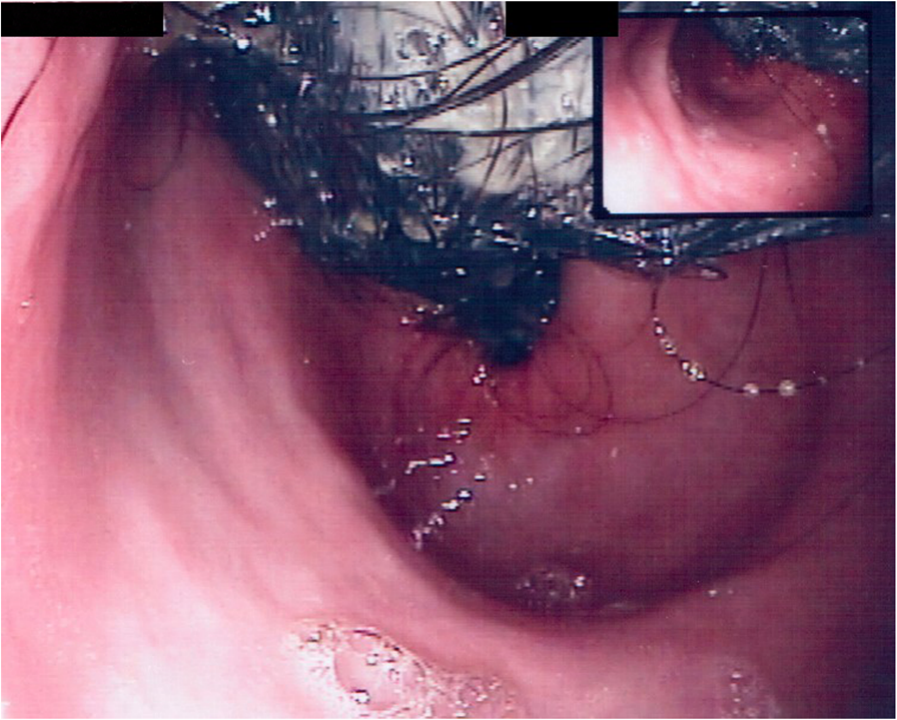
Gastrointestinal endoscopy: large black and hard matter occupying lumen of stomach

**Fig. 3:**
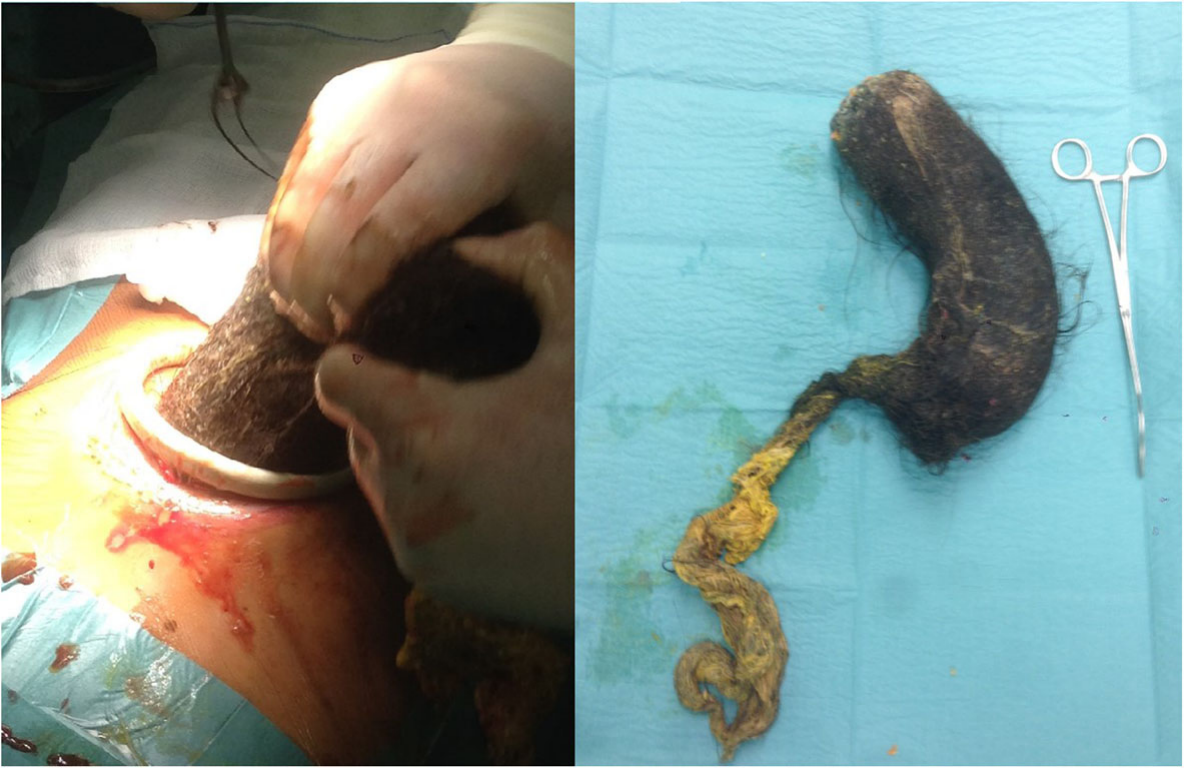
Huge, intra-luminal solid mass excised from the stomach

After appropriate transfusion of erythrocytes and plasma, endoscopist evaluation was requested. In the light of the large size of the mass, it was thought that endoscopy could likely fail to remove the mass **(**Fig. 2**)**. Thus, the patient was referred for surgical evaluation. The patient underwent surgical intervention by means of a median sovraumbilical laparotomy and anterior gastrotomy; a 1360 g trichobezoar was extracted **(**Fig. 3**)** and sent to histological examination which concluded for gastric trichobezoar, with vegetable fibers and amorphous matter.

The post-operative course was characterized by the onset of fever. A CT scan of the abdomen and thorax revealed perisplenic and pleural effusion with pulmonary consolidation which required a broad-spectrum antibiotic therapy and the abdominal placement of a drainage tube which was removed 6 days later. Feeding was gradually reintroduced, first by means of total parenteral nutrition and then orally with supplementation of iron, vitamin B complex, folate and other micronutrients. During this period, the patient was carefully monitored for post surgical complications.

At the time of hospital discharge, blood values were normalized and we observed an initial catch-up weight; pulmonary and abdominal US didn’t show any residual free fluid collection.

The patient underwent both psychiatric and psychological evaluations before discharge. Both concluded for PICA disorder, on the basis of history of reported and chronic ingestion of small stones which started during primary school attendance and subsequent hair ingestion that the patient was pleased to eat because she found them to be tasty. According to DSM-V (Diagnostic and Statystical Manual of Mental Disorders) [5], PICA is characterized by an appetite for substances that are largely non-nutritive, such as ice (pagophagia), hair (trichophagia), paper (papyrophagia), drywall or paint, metal (metallophagia), stones (lithophagia) or earth (geophagia), glass (hyalophagia) or feces (coprophagia). To be considered as PICA, these actions must persist for more than 1 month at an age where eating such objects is considered developmentally inappropriate, not part of culturally sanctioned practice and sufficiently severe to warrant clinical attention. As our patient met all the diagnostic criteria, she has been included into our follow up program for patients affected by Eating Disorders in order to prevent possible recurrence of PICA. We didn’t report bezoar recurrence in our 2 years follow up.

## Discussion and conclusion

Trichobezoar, an under-diagnosed entity, has to be considered in the differential diagnosis of IDA and abdominal pain in pediatric emergency department (8 and 18% incidence respectively in our center), especially if an history of PICA is reported. Key point of our case report is that the prompt diagnosis of trichobezoar in an adolescent presenting with severe IDA, abdominal pain and trace elements deficiency leads to immediate and appropriate treatment. Our case also stresses the importance of gradual re-feeding and close clinical monitoring in order to assess complications related to surgery such as surgical site infection and leakage [6]. An important major point is the need of psychological evaluation and follow up in order to prevent PICA recurrence [1].

Most patients with trichobezoars suffer from psychiatric disorders including trichotillomania and trichophagia. It is estimated that 1 out of 2000 children worldwide suffer from trichotillomania and that 30% of them will also suffer from trichophagia [6]. Only 1% of those who suffer from trichophagia will develop a trichobezoar. Most cases of trichobezoars occur in women, 80% of these occurring in childhood/adolescence. A recent study has suggested increased prevalence at the age of 7–8 years and 11–12.5 years. The onset is often triggered by a reaction to negative emotional states (stress, anxiety) [7, 8].

Patients may be asymptomatic for years. Symptoms develop as the trichobezoar enlarges and begins to cause obstruction and may include abdominal pain, nausea and weight loss [9–11]. There may also be malabsorption and IDA [11]. On examination, a mobile, well-defined mass may be palpable in the epigastrium. Differential diagnosis includes splenic enlargement due to falcemic crisis, left liver lobe tumour, neuroblastoma and carcinoma of the stomach. There are many complications associated with trichobezoars described: gastric ulceration, perforation, haemorrhage and obstruction among these.

US scan reveals highly echogenic areas but is often not diagnostic. CT abdomen is the investigation of choice for conclusive diagnosis and well-defined heterogeneous masses, separate from the gastric lumen wall, are seen. If there is obstruction, dilated loops may also be present [4, 6].

Surgical intervention has been previously reported as the only way for removing a bezoar, nowadays it is indicated only after failure of other conservative treatments such as endoscopy and pharmacological approaches or the recurrence of treatment-related complications [2].

Pharmacological strategies include the fragmentation by using the Cola-treatment with Cola direct injection into the bezoars to solve it or carbonated liquid and enzymatic dissolution with papaine which hydrolyzes the proteins [12].

Endoscopic approaches utilize different devices to obtain the fragmentation of the bezoars such as snares, forceps, baskets, electro-hydraulic lithotripters, needle-knife and argon plasma coagulation; a recent technique reported by *G. Grande* et al. [13] demonstrates the successful use of Ho:Yag laser fragmentation of the mass [14]. Large bezoars are managed only with surgical intervention, by means of laparotomic or even laparoscopic extraction.

Since many of these patients suffer from psychiatric pathologies with emotional problems, mental retardation and eating disorders, psychiatric counselling plays an important role in order to prevent bezoar recurrence. Although studies on the pharmacotherapy of trichotillomania remain inconsistent, some patients seem to respond to fluoxetine or other serotonin reuptake inhibitors [15]. Parental counselling is also a regular part of treatment to prevent recurrence. The patients’ long term prognosis is reported to be excellent if behavioral therapy is used to control trichophagia, and psychological/psychiatric follow-up is maintained.

In summary, the Rapunzel syndrome is an under-diagnosed entity that has to be considered in children and adolescents, expecially female, referring to pediatric emergency department with IDA and abdominal pain, expecially if an history of PICA is reported. Early diagnosis and an appropriate therapy can reduce morbidity and mortality. Gradual re-feeding after surgery allows adequate catch-up weight with normalization of haematochemical nutritional parameters. Psychological counselling plays a pivotal role in order to prevent PICA and related therefore bezoar recurrence.

## Abbreviations

CT: Computed tomography
DSM: Diagnostic and Statystical Manual of Mental Disorders
IDA: Iron deficiency anemia
US: Ultrasound
